# Food Applications and Potential Health Benefits of Pomegranate and its Derivatives

**DOI:** 10.3390/foods9020122

**Published:** 2020-01-23

**Authors:** Panagiotis Kandylis, Evangelos Kokkinomagoulos

**Affiliations:** Laboratory of Oenology and Alcoholic Beverages, Department of Food Science and Technology, School of Agriculture, Aristotle University of Thessaloniki, P.O. Box 235, 54124 Thessaloniki, Greece; ekokkinom@gmail.com

**Keywords:** extract, juice, peel, seed, antioxidant, polyphenols, health benefits, dairy, meat, food

## Abstract

Pomegranate (*Punica granatum* L.) is an ancient fruit that is particularly cultivated in west Asia, though it is also cultivated in the Mediterranean region and other parts of the world. Since ancient years, its consumption has been associated with numerous health benefits. In recent years, several in vitro and in vivo studies have revealed its beneficial physiological activities, especially its antioxidative, antimicrobial and anti-inflammatory properties. Furthermore, human-based studies have shown promising results and have indicated pomegranate potential as a protective agent of several diseases. Following that trend and the food industry’s demand for antioxidants and antimicrobials from natural sources, the application of pomegranate and its extracts (mainly as antioxidants and antimicrobials), has been studied extensively in different types of food products with satisfactory results. This review aims to present all the recent studies and trends in the applications of pomegranate in the food industry and how these trends have affected product’s physicochemical characteristics and shelf-life. In addition, recent in vitro and in vivo studies are presented in order to reveal pomegranate’s potential in the treatment of several diseases.

## 1. Introduction

Pomegranate is the well-established fruit of a shrub (*Punica granatum* L.) that is particularly cultivated in west Asia and in the region around the Mediterranean, as well as other parts of the world, including America, where the climate is suitable for its growth [1]. The shrub normally grows up to 5 m, but in some cases, it may reach a morphology of a tree that is as tall as 10 m, except for dwarf cultivars that grow up to 1–2 m [2]. Climates that simulate that of Mediterranean (with high sunlight-exposed mild winters and dry summers), seem to be ideal for the growth of pomegranate. Most varieties are deciduous, although there have been reports of evergreen and conditionally deciduous pomegranates, depending on the altitude and temperature of the zone. The fruit is categorized as a fleshy berry. Its shape is nearly round, with a diameter up to 10 cm, and there is a crown-shaped calyx at the top. Inside the leathery exocarp is a fleshy mesocarp, organized in chambers that are separated by membranes. The arils contain the edible portion of the fruit. The exocarp, namely the pomegranate peel, comprises around 50% of the whole fruit, while the edible part consists of 10% seeds and 40% arils [3]. The whole pomegranate and its juice have an intense color for which selected bioactive compounds are responsible, especially anthocyanins. Therefore, the variation in color amongst different cultivars is mainly due to the different concentration of these compounds.

The scope of the current review is to point out pomegranate applications in food industry concerning the product’s physicochemical characteristics and shelf life. Moreover, there is an effort to present the pomegranate’s potential against a number of diseases through in vitro and in vivo studies.

## 2. General Description

### 2.1. History

The pomegranate originates in the Middle East, with findings such as fossilized leaves, branches and seeds dating back to the early Bronze Age (3500–2000 BC). Scientists have placed it in the first five positions in the list of the oldest cultivated fruits, along with the olive, grape, date palm and fig [4], while references of pomegranate exist in the Koran and the Bible [5]. In many religions and cultures, pomegranate is thought to be an auspicious symbol, mostly of life, luck, abundance and fertility [6]. The process of the domestication of pomegranate took place during the prehistoric times, when traders, sailors and missionaries are said to have been responsible for the introduction of pomegranate to the Mediterranean region, Mexico and California. Its spread through Eurasia and America demonstrates the versatility of the plant as far as climatic and soil conditions are concerned, and this is actually the reason for the fruit’s current morphological conditions.

### 2.2. Taxonomy

The pomegranate is part of the Punicaceae family and the *Punica* genus, whereas two species exist: *Punica granatum* and *Punica protopunica*. The latter is endemic to Socotra Island (Yemen) and is considered to have played an important role in the evolution of the cultivated form of pomegranate, since it is considered the ancestor of the genus *Punica* [5]. The genus *Punica* has distinctive characteristics that place it in the order Myrtales, although the family under which it should be is debatable [5]. Historically, due to early morphological studies, it is believed that the *Punica* genus should be considered under Lythraceae [2], but there have been many controversies regarding its classification into a family. However, due to its unique morphology and other features, such as fruits with leathery pericarp, pulpy seeds with edible sarcotesta, an ovule with a multilayered outer integument, and a unicellular archesporium, pomegranate has been found to differ from other typical Lythraceae genera and has therefore been included in a separate family, Punicaceae [2]. The first written description of the genus *Punica* dates back to 1753 and belongs to C. Linnaeus [2]. The current scientific name *Punica granatum* can be translated to “seeded apple” (Punica—apple; granatum—grainy) [5]. Due to the plant’s high tolerance to drought conditions, pomegranate is considered a suitable option for the cultivation of fruit crops in arid zones.

### 2.3. Cultivars

There are many different cultivars of pomegranate (more than 500) spread all over the world. However, the type of cultivars that have prevailed in certain regions reflects the preferences and taste of the local populations, e.g., nonacidic cultivars are favored in India. The origin of the current cultivars is covered by a shroud of doubt because most of them are derivatives of mutations with no recordings of their origin. Exemptions are some cultivars that are the result of deliberate breeding.

In general, the same basic pomegranate fruit is known by different names in different regions, and this is mainly due to the fact that husk and aril color can markedly vary when grown in different regions. These differences mainly affect fruit size, husk color (ranging from yellow to purple, with pink and red most common), aril color (ranging from white to red), seed hardness, maturity, juice content, acidity, sweetness and astringency [7].

Some of the most important cultivars around the world are presented in Table 1.

### 2.4. Composition

**Figure 1:**
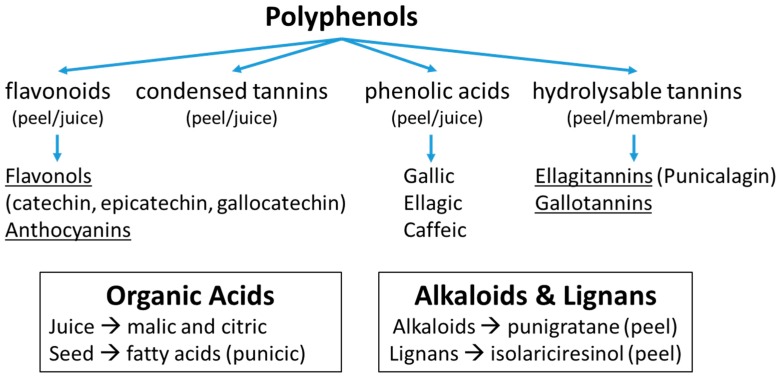
Major polyphenols, organic acids, alkaloids, and lignans of the pomegranate fruit.

Pomegranate is a well-known source of valuable nutritional substances. It contains hydrolysable tannins, condensed tannins, flavonols, anthocyanins, and phenolic and organic acids (Figure 1); compounds that have been studied and related with numerous health benefits against diseases [23]. In addition, it is characterized by a low pH value (usually <4.0), a relatively high acidity (even up to 20 g of citric acid/L of juice), and a sugar content (mainly fructose and glucose) of 70–180 g/L. The exact composition of the fruit depends on many factors, such as the cultivar, soil condition, climate, ripening stage, cultivation techniques, processing conditions, and storage conditions (Table 2).

The edible part of the fruit is at least 50% of the fruit (40% arils and 10% seeds), and the rest is the non-edible peel. Peels are source of phenolics, minerals and complex polysaccharides, while arils, apart from water (85%), contain sugars, pectin, organic acids, phenolics, and flavonoids—principally anthocyanins. Seeds contain proteins, crude fibers, vitamins, minerals, pectin, sugars, polyphenols, isoflavones, and the oil that is derived from them (12–20%) is characterized by a high content of polyunsaturated fatty acids such as linolenic and linoleic acids, as well as other lipids such as punicic acid, oleic acid, stearic acid, and palmitic acid [24].

The family of hydrolysable tannins contains two members, ellagitannins and gallotannins, which can be hydrolyzed into ellagic and gallic acid, respectively. On the one hand, ellagitannins are mostly present in the pericarp, seeds, flowers, and bark, while, on the other hand, gallotannins are mostly present in the leaves. Punicalagin, a substance belonging in the family of ellagitannins, is responsible for more than the half pomegranate juice’s antioxidant effect [25]. Following the digestion path, ellagitannins are converted by the intestinal flora into urolithins. Other substances that are present in pomegranate juice are phenolic acids, mainly gallic acid and ellagic acid (which belong to hydroxybenzoic acids), as well as caffeic acid, chlorogenic acid, and p-coumaric acid (which belong to hydroxycinnamic acids) [24].

Another constituent that plays a major role in the pomegranate as a functional food is anthocyanins. These water-soluble plant pigments belong to the family of flavonoids and are responsible for the color of the fruit and its juice. They have been thoroughly studied for their numerous effects on health, such as their antioxidant, anti-inflammatory and antiproliferative properties, meaning that they can contribute to the prevention of several diseases [26]. Flavonoids, including flavonols, anthocyanins and phenolic acids, are mainly found in the peel and juice of pomegranate.

It has been reported that the main phenolic compounds in pomegranate juice are anthocyanins, whereas the main phenolic compounds that are found in the mesocarp and pericarp are hydrolysable tannins [27]. It has also been reported that the pomegranate peel has a higher antioxidant capacity than the arils and seeds of the fruit [28], thus making it a potent source of bioactive compounds. This is in accordance with other studies that have indicated that the pomegranate peel has a higher concentration of phenolic compounds in comparison with pomegranate juice [29].

The bioactive health effects of pomegranate have been attributed to the broad range of phytochemicals that it contains. The most predominant phytochemical in pomegranate, as was already described, is considered to be polyphenols—mainly hydrolysable ellagitannins and anthocyanins [24]. However, it has been proven that there is a synergistic effect among compounds that further increases their bioactivity. For example, quercetin and ellagic acid show better inhibition properties against cancer cell growth in comparison to each of the substances alone [33].

Usually, the characterization of a matrix, and especially of the compounds to be studied, is based on previously published works. Nowadays, along with advancements in technology, it is possible to create databases with properties of known substances. An example of this is high resolution mass spectroscopy, where the analyzed substance can be identified based on a database that has been constructed and allows for the identification of new compounds beyond those already known [34]. A recent study [35] characterized pomegranate aril anthocyanin extracts by high pressure liquid chromatography (HPLC) coupled with high resolution mass spectroscopy (HRMS), and the five most dominant anthocyanins were delphinidin-3,5-diglucoside, cyanidin-3,5-diglucoside, pelargonidin-3,5-diglucoside, delphinidin-3-glucoside, and cyanidin-3-glucoside.

## 3. Health Benefits

Fruits, in general, play a major role in the maintenance of a balanced diet. They provide plenty of macro- and micronutrients, as well as bioactive compounds that promote health. Over the past few decades, there have been many studies indicating the importance of fruit consumption in the prevention of health-associated risks, as well as campaigns for the incorporation of fruit in the diets of children.

Numerous studies concerning the potential health benefits, in addition to the nutritional value, of the pomegranate and its constituents have been conducted. Encouraging findings have increased the interest shown in this specific fruit over the past few years. Pomegranate, being rich in bioactive compounds like polyphenols, has shown many health-related properties, such as antioxidant, anti-inflammatory and antihypertensive, through in vivo and in vitro studies. The health-promoting properties of the fruit are considered to mainly be due to the presence of punicalagin and, to a lesser extent, to other metabolites, such as flavonols and anthocyanins [36]. Several studies have brought up the potential contribution of pomegranate in the treatment of cancer, diabetes and heart disease.

### 3.1. In Vitro Studies

Several in vitro studies have been conducted by mainly using pomegranate juice and extracts in order to associate them with numerous health benefits (Table 3).

#### 3.1.1. Prebiotic Effect and Antimicrobial Activity

One of the most important health-related functions of the pomegranate and its derivatives is their effect on gut microbiota and their potential use as antimicrobial agents. It is well known that ellagitannins, the most abundant group of polyphenols in pomegranate, are hydrolyzed in ellagic acid in the gut before being further metabolized by the colon microbiota to form urolithin A and B [38]. This has been associated with the prebiotic potential of the pomegranate and its products. Indeed, in an in vitro study with fecal bacteria, pomegranate by-products enhanced the growth of *Bifidobacterium* spp. and *Lactobacillus* spp. acting as a prebiotic [59]. Pure cultures of *Bifidobacterium* and *Lactobacillus* strains have been proven capable of utilizing ellagic acid and glycosyl ellagic acid [40]. Pomegranate by-products, such as gallic acid, ellagic acid and glucose units, are used by fecal bacteria to produce urolithins and increase the production of short chain fatty acids like acetate, propionate and butyrate [59]. In a similar study, pomegranate juice and extracts were used in in vitro stool cultures and were proven to enhance the growth of *Bifidobacteria* and *Lactobacilli* while simultaneously inhibiting the growth of the *Bacteroides fragilis* group, *Clostridia*, and Enterobacteriaceae [40]. Furthermore, pomegranate by-products and punicalagins were found to inhibit the growth of pathogenic *Clostridia* and *Staphyloccocus aureus* in human gut bacteria cultures [60]. These results may reveal the potential prebiotic activity of pomegranate juice and extracts on human gut microflora. The prebiotic effect of pomegranate juice has been evaluated by using the simulated gastrointestinal digestion of different pomegranate juices with lactic acid bacteria, resulting in the increased bio-accessibility of phenolic compounds and ensuring the survival of lactic acid bacteria (which may be due to metabolism of the ellagitannins, epicatechin, and catechin) [61].

#### 3.1.2. Anticarcinogenic Effect

Pomegranate extracts have also been evaluated for their anticarcinogenic activity against numerous cancer types. More specifically, pomegranate extracts have been shown to block nuclear factor kappa B (NF-κB) activity in a prostate cancer model [62] and renal cell carcinoma [43] in vitro. Therefore, pomegranate extracts may be used as dietary adjuncts to manage patients with small, localized, incidentally identified renal tumors, and this may lead to the avoidance of nephrectomy [43]. Pomegranate peel extracts and punicalagin, a polyphenol from pomegranate fruit, have shown growth inhibition on prostate cancer cells and anti-proliferative activity via the induction of apoptosis [45,51]. Additionally, in vitro studies have revealed that punicalagin induces the cell death of papillary thyroid carcinoma cells [54], inhibits cell proliferation in a non-small lung carcinoma cell line [52], and exerts a strong anti-proliferative activity against the human lung, breast, and cervical cancer cell lines [53]. The pomegranate and its products have been associated with the prevention of cancer metastasis. In a recent study, the main molecular targets of pomegranate that are associated with cancer metastasis were reviewed [63]. These targets include (i) molecules that are involved in cell–cell and cell–extracellular matrix adhesions, (ii) pro-inflammatory and pro-angiogenic molecules, (iii) modulators of cytoskeleton dynamics, and (iv) regulators of cancer cell anoikis and chemotaxis. Furthermore, the antimetastatic effect of pomegranate may be attributed to molecular changes in the extracellular matrix.

#### 3.1.3. Skin Health

Pomegranate phenolics may be used as natural antioxidants for cosmeceutical applications for skin health, as a recent in vitro study showed their protective effects against H_2_O_2_-induced oxidative stress and cytotoxicity in human keratinocyte HaCaT cells [37]. In addition, pomegranate products (juice, extract and oil) that are derived from the remaining material after pomegranate fruit squeezing for juice production have presented photo-chemopreventive effects [57]. More specifically, pomegranate products have been shown to inhibit UVB-mediated DNA and protein damage, increased proliferating cell nuclear antigen and tropoelastin levels along with the degradation of extracellular matrix proteins in human reconstituted skin.

#### 3.1.4. Obesity, Diabetes, Alzheimer’s Disease, Osteoporosis and Dental Health

In recent years, obesity has become a worldwide health problem, and several studies have focused on it. Pomegranate juice and some specific components, like ellagic acid and punicalagin, have presented the ability to inhibit amine oxidases, α-glucosidase, dipeptidyl peptidase-4, lipase, triglyceride accumulation, and adipogenesis-related genes, as well as to decrease lipogenesis and lipolysis in mouse and human adipose cells. These results have shown the great potential of pomegranate juice and its components to be used as a functional food for the prevention of diseases that are associated with obesity, diabetes and dyslipidemias [41,42]. In addition, pomegranate juice extract and ellagitannins have been shown to inhibit α-glucosidase activity in vitro and to reduce starch digestibility under simulated gastrointestinal conditions, confirming the great potential of pomegranate juice to improve postprandial hyperglycemia, which is linked to type II diabetes [39].

Several studies have proposed the use of pomegranate, and especially its derivative punicalagin and urolithins, as a potential nutritional strategy in slowing the progression of neurodegenerative disorders such as Alzheimer’s disease [64]. Urolithins inhibit neuroinflammation [58], while punicalagin inhibits lipopolysaccharide-induced memory impairment via anti-inflammatory and anti-amylogenic mechanisms [55].

Pomegranate extracts (peel and fruit) stimulate osteoblastic differentiation [48,49], while punicalagin attenuates osteoclast differentiation in vitro [56]; therefore, pomegranate juice or extracts might be useful as agents for the treatment of osteoporosis. In addition, the regular consumption of pomegranate may benefit the skeletal tissues of the host [49].

Pomegranate has also been proposed for the maintenance of dental health. *Streptococcus mutans* is one of the major microorganisms of dental flora, and it is capable of producing acids, soluble extracellular polysaccharides, and insoluble extracellular polysaccharides, as well as forming biofilms. An in vitro study showed that pomegranate peel extracts are capable of inhibiting the growth of cariogenic bacteria at high concentrations (up to 12.5–25.0 mg/mL); however, these concentrations are difficult to maintain in the oral cavity because of the constant saliva flow. In lower concentrations, these extracts have been shown to inhibit biofilm formation, acid production, and extracellular polysaccharides production by *S. mutans*, showing these extracts’ potential to prevent dental caries [46]. In another study, pomegranate peel and juice extracts presented inhibitory effects, not only against *S. mutans* but also against *Rothia dentocariosa*, which has been found on the carious lesions of human teeth and may cause several diseases like endocarditis, pneumonia and infections of the peritoneum and lung [47].

### 3.2. Studies Using Mice Models

Apart from in vitro studies that have used pomegranate juice and extracts, there have also been several studies that have used mice models (Table 4).

#### 3.2.1. Obesity, Diabetes

The pomegranate and especially its extracts have attracted the attention of the research community due to their numerous health benefits, some of them associated with obesity. Several mouse intervention studies have demonstrated that pomegranate extracts can decrease inflammation and LDL (low-density lipoprotein) cholesterol in high-fat diet-induced obese mice [76,77] and can reduce hepatic lipid peroxidation and serum glucose levels in healthy rats, in addition to improving glycemic control and increased relative beta cell number in alloxan-induced diabetic rats [78,79]. In a recent study, the combination of pomegranate extracts with inulin led to enhanced cholesterol-lowering effects [80]. In addition, in the same study, the mechanism of the pomegranate extracts’ action was revealed—more specifically, they lowered cholesterol by increasing bile acid synthesis. Furthermore, punicalagin reduces the high-fat diet-induced accumulation of cardiac triglyceride and cholesterol in obese rats via adenosine monophosphate (AMP)–activated protein kinase (AMPK) activation [81]. Finally, pomegranate leaf extracts can inhibit lipid absorption and reduce blood triglycerides and total cholesterol in hyperlipidemic mice by inhibiting lipase activity [82].

#### 3.2.2. Prevention and Treatment of Infections

The pomegranate and its products have been evaluated as agents for the prevention and even treatment of several bacterial or virus infections in mice model systems. Coccidiosis, the most prevalent disease, especially in poultry farms, causes widespread economic loss. The use of pomegranate peel extracts on the outcome of coccidiosis in mice has been found to attenuate inflammation and injury of the jejunum that is induced by *Eimeria papillata* infections [83]. In a similar study, treatment with a pomegranate peel extract decreased the pathogenicity of *Citrobacter rodentium* infections in mice, suggesting an alteration of the microbiome, making it more resistant to *Citrobacter rodentium* [82]. *Citrobacter rodentium* mimics many aspects of human enteropathogenic *Escherichia coli* infections and is therefore used in several studies with mice. A pomegranate peel extract (containing punicalin, punicalagin, and ellagic acid) reduced *Citrobacter rodentium* infection-induced weight loss and colon damage that correlated with a decreased mortality and reduced colonization of the spleen [84]. In addition, a pomegranate peel extract proved to be valuable in the prevention and treatment of *Giardia lamblia* infection (giardiasis) of the human small intestine [75]. These studies indicate that pomegranate polyphenols may mitigate the pathogenic effects of food-borne bacterial pathogens.

#### 3.2.3. Other Health Benefits

The addition of pomegranates in the diet may slow the progression of cognitive and behavioral impairments in Alzheimer’s disease [65], while pomegranate extracts have been shown to have anti-inflammatory and antioxidant effects on cecal ligation and puncture-induced acute liver injury [66]; they have also been shown to protect against arsenic-induced inflammation and apoptosis in the liver cells of male Swiss albino mice [67]. Another important product of the pomegranate with several health benefits is the pomegranate peel polysaccharides (rhamnose, glucuronic acid, galacturonic acid, glucose and xylose), which may be used in efficacious adjacent immunopotentiating therapy or an alternative means in lessening chemotherapy-induced immunosuppression; they can also be utilized as immunostimulants for the food and pharmaceutical industries [68]. Several studies have shown that pomegranate peel polysaccharides may enhance the immunomodulatory effect, induced by cyclophosphamide, of immunosuppressed mice [68], exhibit a strong protective effects against CCl_4_-induced liver injury in mice [69], and, at low doses, alleviate contact hypersensitivity symptoms, suggesting that they may provide beneficial effects on allergic contact dermatitis at physiologically relevant doses in humans [70].

### 3.3. Human Studies

The significance of pomegranate health benefits has been revealed by its adaption in several human-based studies (Table 5). The consumption of pomegranate juice for a period of eight weeks showed beneficial effects on blood pressure, serum triglycerides, high-density lipoprotein cholesterol, oxidative stress and inflammation in hemodialysis patients [85]. In patients with type 2 diabetes, a consumption of 1.5 mL/kg body weight reduced serum erythropoietin level after three hours [86], while a 200 mL/day consumption for six weeks reduced systolic and diastolic blood pressure without affecting the lipid profile [87]. In addition, the daily consumption of pomegranate juice (230 mL) has been associated with the stabilization of the ability to learn visual information over a 12 month period [88]. The consumption of pomegranate juice has also been proposed to athletes, and a systematic study for a 21 day period showed an improvement in malondialdehyde and carbonyls levels, and, thus, a decrease of the oxidative damage caused by exercise [89]. Finally, pomegranate juice has been associated with a reduction of inflammation, muscle damage, and an increase of platelets blood levels in healthy people [90]. A 30 day supplementation with pomegranate extracts in individuals with overweight and obesity beneficially affected body weight, serum glucose, insulin, triglyceride, total cholesterol, LDL–C, HDL–C (high-density lipoprotein–cholesterol) and LDL–C to HDL–C proportion, while also acting as an anti-inflammatory agent, lowering inflammatory and lipid peroxidation biomarkers [91]. An eight week supplementation with pomegranate peel extracts attenuated the systolic and diastolic blood pressure in patients with type 2 diabetes and also presented hypolipemic, hypoglycemic, and antioxidative potential [92]. Finally, clinical studies with pomegranate seed oil in type 2 diabetic patients have resulted in reductions in the levels of fasting blood sugar, interleukin-6 and TNF-α (tumor necrosis factor-α); however, no significant changes have been observed in insulin and lipid profiles [93,94].

## 4. Applications in Food Products

The pomegranate, as has been mentioned above, is considered to be a fruit with many beneficial properties that mainly affect health. In addition, many of its sensory properties, such as color and aroma, present great interest in the food industry and can be utilized in many ways. Many researchers have studied the effect of pomegranate addition in the properties of several foods products, such as dairy products (Table 6), films and coatings for food packaging (Table 7), meat and fish products (Table 8), and cereal and nuts products (Table 9).

### 4.1. Dairy Products

Pomegranate juice, in the form of powder, may be used in yogurt production, mainly as a replacer of sucrose content. The addition of pomegranate juice powder (5%) has been shown to lead to a product with an increased total phenolic content, an increased antioxidant activity, and a higher in vitro bio-accessibility. Furthermore, it has also been shown to positively affect the sensory characteristics of the product, resulting in a more solid-like behavior mainly due to phenolic–protein interactions [98]. In a similar study with kefir-like products, the addition of fresh juice (5%) resulted in products with an increased viscosity and acidity; however, the addition of honey was necessary in order to improve the sweetness [97].

The addition of a pomegranate peel extract is another way to add the beneficial properties of pomegranate to dairy products. This extract is mainly used to increase the antioxidant activity of the products, as well as their storage shelf life [100,101]. In addition, it has been used in cheese production and has resulted in improved lipid oxidative stability and storage quality [99]. However, the addition of this extract may lead to negative alterations of the sensory characteristics of the products, mainly due to its high astringency and bitterness [101]. In general, the pomegranate peel extract may be used as a promising natural preservative in fermented dairy products, though it should be used in low concentrations in order to avoid the adversarial effect on sensory attributes. The addition of honey may reduce these effects and improve the acceptability of these products, as in the case of freeze-dried yogurt [102].

Pomegranate seed powder, which is rich in conjugated linolenic acids, has also been incorporated in dairy products like yogurt. Compared to a control, yogurt enriched with 0.5% (w/v) pomegranate seed powder showed similar nutritional and pH values, higher antioxidant activities, desirable fatty acid and conjugated linolenic acids contents, and lower atherogenicity indexes [103].

### 4.2. Films and Coatings

Nowadays, there is an increase of interest for the development of novel food packaging materials and, especially, edible packaging materials. Pomegranate components have been used in the development of such products due to their increased phenolic content and antioxidant properties. Several films and coatings have been developed by incorporating pomegranate peel extracts. A zein-based film has been produced by incorporating pomegranate peel extracts at concentrations up to 75 mg/mL of film [104]. The addition of pomegranate peel extracts leads to the increased tensile strength, elongation at break, total phenolic content and antioxidant activity of zein films, whereas film solubility and water vapor transmission rate decrease and thickness remains constant. This film has been shown to present inhibitory activity against several pathogenic bacteria and has been used for the packaging of cheese, inhibiting the growth of spoilage microorganisms but not affecting lactic acid bacteria. In addition, cheese, with zein-pomegranate packaging, has low protein and lipid oxidation products during storage compared to cheese that is packaged with zein film without pomegranate peel extracts.

Chitosan is a cationic polysaccharide that is obtained by the deacetylation of chitin, and, due to its biodegradability, biocompatibility, antimicrobial activity and non-toxicity, is considered a very promising and eco-friendly material for different purposes, including food coatings systems [105]. In addition, pomegranate peel extracts have been proven capable of improving the functional characteristics of chitosan-based materials by enhancing the desired properties for their potential application as food coatings [105]. Chitosan-based coatings with incorporated pomegranate peel extracts have been used in several products, such as rainbow trout [106], pacific white shrimp [107] and strawberries [108]. In all studies, an improvement in sensory characteristics of the final product has been reported in combination with an extension of shelf life. More specifically, in the case of pacific white shrimps, an inhibition of melanosis has been reported, while a decrease of lipid and protein oxidation has been reported in rainbow trout.

Pomegranate peel powder has also been used for the preparation of edible films based on starch [109]. The powder has been used as an antimicrobial and reinforcing agent. The new film has presented inhibitory actions against both *Staphylococcus aureus* and *Salmonella*. The addition of pomegranate peel powder in a starch based matrix has also exhibited better mechanical properties by enhancing the stiffness, modulus, tensile strength and drop impact strength of the matrix. Another edible coating material based on chitosan or/and locust bean gum with an incorporated pomegranate peel extract was developed to control the growth of *Penicillium digitatum* and to reduce the postharvest decay of oranges [110]. The results showed that the addition of a pomegranate peel extract reduced disease incidence by up to 49% on oranges that were artificially inoculated with *P. digitatum*. Pomegranate peel extracts have also been used to formulate surimi-based edible films with superior mechanical and water barrier properties and improved thermal stability for food packaging [111]. The antioxidant activity of bitter vetch seed protein edible films was increased with the addition of pomegranate juice to the film, which formed a solution that resulted in a material with great potential in the active packaging of food systems [112]. Apart from antioxidant activity, the presence of pomegranate juice has also been shown to affect some physicochemical properties of films. More specifically, the films have shown higher total soluble matter, elongation at break, and water vapor permeability, as well as a lower tensile strength, in comparison with control films prepared in the absence of pomegranate juice. In addition, film morphology has been shown to markedly change, with the film surface becoming considerably smoother and with a high number of pores. The interactions in the film forming solution between bitter vetch proteins and the phenolic compounds that are contained in pomegranate juice may be responsible for the observed changes in film properties.

### 4.3. Antimicrobial and Antifungal Agent in Fruits and Juices

The great antimicrobial activity of pomegranate has attracted the interest of researchers to use the fruit as a natural antimicrobial agent or even a preservative in fruits and their juices. In vitro studies of pomegranate peel extracts have revealed their strong antifungal activity against *Botrytis cinerea*, *Penicillium digitatum* and *Penicillium expansum* [124]. Furthermore, when used in artificially inoculated fruits, pomegranate has proven very effective in inhibiting *P. digitatum* and *Penicillium italicum* in lemons, *P. italicum* in grapefruits, and *P. expansum* in apples. However, pomegranate extracts have also been used in fruit juice due to their antimicrobial properties. For example, a commercial pomegranate extract (POMELLA^®^, PE) has successfully been used against *Alicyclobacillus acidoterrestris* cells and spores in apple juice [125].

### 4.4. Meat and Fish Products

Meat products are very susceptible to undesirable alterations during processing and storage, which result in extensive flavor changes, color loss, and protein structure damage, all of which reduce sensory parameters and consumer acceptability [126]. All these undesirable changes are mainly caused by three different biochemical pathways—lipid oxidation, protein decomposition, and microbial contamination—that are more pronounced in minced meats [127]. Therefore, pomegranate has been evaluated as an additive in meat products in order to suppress the development of these effects [128].

Lyophilized pomegranate peel nanoparticles, which have a high phenolic content and antioxidant capacity, were evaluated as antioxidant and antimicrobial additives (up to 1.5%) in meatballs during storage at 4 °C for up to 15 days. The results demonstrated that lyophilized pomegranate peel nanoparticles were more effective in retarding lipid oxidation and improving the microbial quality and cooking characteristics of meatballs compared to samples with 0.01% butylated hydroxytoluene (BHT) and without any treatment [113]. Furthermore, these samples were more sensorially acceptable. In a similar study, the use of pomegranate peel powder (up to 3%) was proven to be effective as a natural preservative in producing high quality beef sausage samples during a storage period of 12 days at 4 °C [114]. The addition of pomegranate peel powder caused a high storage stability and reduced values of the thiobarbituric acid and total volatile nitrogen of prepared beef sausage samples during refrigerated storage. The microbiological criteria of the prepared beef sausage samples with pomegranate peel powder were also improved. In addition, improvements of cooking characteristics, e.g., cooking loss, cooking yield, change in diameter, and change in length, were also reported. A high-ellagic acid commercial pomegranate powder has also been used to reduce the heat resistance of *Escherichia coli* O104:H4 in ground chicken [115]. Indeed, the time to reach a 5.0 log reduction reached a minimum at a pomegranate powder concentration of 1%, producing a 50% decrease in lethality time in comparison to that without added pomegranate powder. This result, in combination with the reported inhibition of formation of carcinogenic aromatic amines by a pomegranate seed extract [129], implies that pomegranate formulations might concurrently inhibit both pathogens and heterocyclic amines in processed meat and poultry products.

Pomegranate peel extracts could potentially be used as natural antioxidant and antibacterial agents in fish products. For example, when shrimps were soaked in methanolic pomegranate peel extracts for 15 min, melanosis, microbial growth, and lipid oxidation could be retarded for up to six days of refrigerated storage [116]. The inhibitory activity followed a dose-dependent manner. In another study, fish patties made with natural extracts, including pomegranate, showed a lower lipid oxidation and, as a result, had an extended shelf life for 11 days of storage under retail display conditions [117]. Furthermore, pomegranate presented a high inhibition against *Listeria monocytogenes*.

### 4.5. Cereal and Nuts Products

Pomegranate peel extracts, either in crude or encapsulated form, were mixed with hazelnut paste in order to extend the shelf life of the product through the inhibition of lipid oxidation [119]. An inhibition of lipid oxidation with a reduced formation of peroxides and a limited solubility of the crude extract in the high lipid content matrix of hazelnut paste were reported. In a similar study, pomegranate peel extracts, which were encapsulated by spray-drying when using orange juice industry by-products as wall materials, were used to fortify cookies with an increased phenolic content and oil oxidation stability [120]. The antioxidant activity of the enriched cookies remained at high levels throughout the whole storage time, and they were preferred for their color and odor by the panelists during sensory evaluation.

Pomegranate seed powder was incorporated in gluten-free bread in order to increase its total phenolic content and antioxidant activity [121]. The results showed that pomegranate seed powder increased the specific volume and springiness of gluten-free breads, whereas their hardness and chewiness decreased significantly with increasing powder additions. In addition, decreases of lightness and yellowness of crumb and crust color, as well as an increase of redness, were reported. In general, the optimum gluten-free bread with the best physical characteristics and high antioxidant properties was found with the use of 7.5% of pomegranate seed powder. Following that trend, pomegranate seed powder has also been used for the production of gluten-free cake [122] and gluten-free (GF) sheeted pasta [123]. Gluten-free cake (containing 25.75% pomegranate seed powder and 0.97% transglutaminase) showed higher total antioxidant activity, ash, fiber, protein and moisture contents, as well as a lower peroxide value, volume index and porosity [122]. In the case of sheeted pasta, an increase of the antioxidant activity reported, but the addition of pomegranate seed powder affected cooking and textural parameters. In general, the lowest concentration of pomegranate seed powder had the lowest effect, and, therefore, gluten-free pasta that was incorporated with up to 7.5% of pomegranate seed powder resulted in a good acceptability [123].

## 5. Conclusions and Future Perspectives

In recent years, the food industry’s demand for antioxidants from natural sources has continuously grown, especially now with the increased numbers of adverse toxicological reports on many synthetic compounds. Therefore, pomegranate, which presents extremely high antioxidant and antimicrobial properties, has a great potential for applications in food products. The application of pomegranate and its extracts, mainly as antioxidants and antimicrobials, has been extensively studied in different types of food products and has shown very promising results. In addition, many studies have shown that these additives can positively affect the overall sensory quality, and hence the shelf life, of food products.

Though pomegranate is a food and is therefore regarded as safe, this judgement is not applied to its extracts, such as its peel and seed extracts, and further research is needed to determine their safe limits [128]. Therefore, several studies have been carried out and must continue to be carried out in order to ascertain the safe use of these edible natural compounds.

On the other hand, pomegranate juice and the wide variety of compounds derived from it have been the focus of many in vitro and in vivo studies that have revealed their beneficial physiological activities, especially their antioxidative, antimicrobial and anti-inflammatory properties. These studies have concluded that the regular consumption of pomegranate fruit, juice, or even its compounds added in other food products acts beneficially for one’s health and may even protect against or improve the course of several diseases like obesity, diabetes, cardiovascular diseases, and even some cancer types.

Future studies should be focused on the identification of the mechanisms that are associated with the previously mentioned activities of pomegranate and its products, its possible synergistic effects with other compounds of foods, and, most importantly, its possible interactions with the gut microflora of hosts. All these studies will provide all the necessary scientific evidence that is required in order to fully understand the potential of pomegranate as a source of natural food preservatives and a therapeutic agent.

## Figures and Tables

**Table 1:** Some of the pomegranate varieties around the world.

Country	Variety	References
China	Dabaitian, Heyinruanzi, Tongpi, Bopi	[8]
Egypt	Arabi, Manfaloty, Nab ElGamal, Wardy	[9]
Georgia	Pirosmani, Rubin, Shirvani, Slunar, Vedzisuri, Imeretis Sauketeso	[10]
Greece	Hermione, Persephone, Porphirogeneti	[11]
India	Ganesh, Mridula, Bhagwa, Ruby, Alandi	[12,13]
Iran	Malas-e-Saveh, Rabab-e-Neyriz, Malas-e-Yazdi, Sishe Kape-Ferdos, Naderi-e-Budrood	[14]
Israel	Rosh Hapered, Malisi, Wonderful, Asmar	[13,15]
Italy	Dente di Cavallo, Neirana, Profeta, A dente Molfetta, Ecotipo Turi, Maddaloni Dolce, Giardino Chiuso Dolce	[16,17]
Malta	Blance, Dulce Colourada, Cagin	[13,18]
Morocco	Gjebali, Djeibi, Grenade Jaune, Grenade rouge, Bzou, Sefri, Chelfi	[19]
Spain	Mollar de Elche, Agri de albatera, Valenciana	[13,20]
Tunisia	Gabsi, Tounsi, Zehri, Mezzi, Jebali, Garoussi, Kalaii, Zaghouani	[10,21]
Turkey	Cekirdksiz, Ernar, Fellahyemez, Hatay, Akanar, Hicaznar, Janarnar	[10,13]
USA	Wonderful, Early Foothill, Granada, Spanish sweet, Ruby red	[13,18,22]

**Table 2:** Physicochemical characteristics of pomegranate varieties’ juices.

Characteristic	Sweet ^1^ Varieties	Sour–Sweet ^1^ Varieties	Sour ^1^ Varieties	Wonderful ^2^ Variety	Bhagwa ^3^ Variety
TSS ^4^ (°Brix)	10.0–16.5	12.0–15.0	13.0–16.0	15.7–17.5	16.2 ± 0.2
pH	4.0–4.2	3.6–3.7	2.9–3.6	2.8–3.6	3.6 ± 0.1
TA ^5^ (g/L)	4.0–6.8	8.2–11.4	14.8–24.5	11.0–13.0	3.8 ± 0.2
Fructose (%*w/v*)	4.1–6.0	3.9–4.0	3.5–4.0	7.8–9.1	8.2
Glucose (%*w/v*)	4.3–6.4	4.3–4.4	3.4–3.9	7.3–8.4	7.0
Total sugars (%*w/v*)	8.5–12.4	8.3–8.4	7.2–7.9	15.3–17.5	15.2

^1^ Several varieties from Iran [30]; ^2^ [31]; ^3^ [32]; ^4^ Total soluble solids; ^5^ Titratable acidity (g citric acid/L of juice).

**Table 3:** Recent health-related pomegranate in vitro studies.

Derivative	Effect	References
whole fruit extract	↓H_2_O_2_-induced oxidative stress; ↓apoptosis; natural antioxidants for skin health	[37]
whole fruit extract	antimicrobial activity against 29 clinical *Clostridium difficile* isolates	[38]
juice extract	inhibition of a-glucosidase activity	[39]
extract and juice	prebiotic effect	[40]
juice	↓lipogenesis and lipolysis	[41]
juice	inhibition of lipase, α-glucosidase and dipeptidyl peptidase-4	[42]
peel extract	inhibition of renal cell carcinoma growth	[43]
peel extract	anti-neurodegenerative	[44]
peel extract	↑apoptosis and ↓metastasis in prostate cancer cells	[45]
peel and juice extract	inhibition of cariogenic bacteria	[46,47]
peel and fruit extract	stimulates osteoblastic differentiation (osteoporosis)	[48,49]
peel polysaccharide	immunostimulatory effect	[50]
punicalagin	antiproliferative activity against human lung, breast, cervical and prostate cancer cells	[51,52,53]
punicalagin	↑papillary thyroid human carcinoma cell death	[54]
punicalagin	inhibition of lipopolysaccharide-induced memory impairment (Alzheimer’s disease)	[55]
punicalagin	attenuates osteoclast differentiation (osteoporosis)	[56]
pomegranate-derived products (juice, extract, oil)	photo-chemopreventive effect in human reconstituted skin	[57]
urolithins	inhibition of neuroinflammation (Alzheimer’s disease)	[58]

**Table 4:** Recent health-related pomegranate studies that used mice models.

Derivative	Effect	References
fruit	↓of progression of cognitive and behavioral impairments in Alzheimer’s disease	[65]
whole fruit extract	anti-inflammatory and antioxidant effects	[66]
whole fruit extract	↓apoptosis and inflammation in liver cells	[67]
peel polysaccharide	↓weight loss and ↑immune organ index of immunosuppressed mice	[68]
peel polysaccharide	protection against CCl_4_-induced liver injury	[69]
pomegranate aril extract	inhibition of contact hypersensitivity of allergic dermatitis	[70]
juice	↑hypoxia-induced fetal growth and ↓apoptosis in the placenta in pregnant mice	[71]
juice	neuroprotection and protection against oxidative damage in Parkinson’s disease rat model	[72]
juice	antileishmanial activity, probably by boosting the endogenous antioxidant activity in female BALB/c mice	[73]
leaf	↓total serum cholesterol and triglycerides of hyperlipidemic mice	[74]
peel extract	contribution in prevention and treatment of *Giardia lamblia* infection	[75]
peel extract	preventing bone loss associated with ovariectomy in mice	[48]

**Table 5:** Recent health-related pomegranate studies in humans.

Derivative	Subject	Effect	References
whole fruit extract	overweight and obese patients	anti-inflammatory effect; ↓body weight, serum glucose, total cholesterol, LDL; ↑HDL	[91]
juice	hemodialysis patients	improved blood pressure, serum triglycerides, HDL, oxidative stress and inflammation	[85]
juice	humans with type 2 diabetes	↓serum erythropoietin level	[86]
juice	humans with type 2 diabetes	↓systolic and diastolic blood pressure	[87]
juice	healthy adults	maintains visual memory skills	[88]
juice	endurance-based athletes	modulation of fat and protein damage	[89]
juice	active healthy men	↓systolic blood pressure, creatinine and muscle damage parameters	[90]
seed oil	humans with type 2 diabetes	↓levels of fasting blood sugar	[93,94]
peel extract	humans with type 2 diabetes	hypolipemic, hypoglycemic, and antioxidative potential	[92]
peel extract	patients with dyslipidemia	↓systolic blood pressure, LDL, total cholesterol; ↑HDL	[95]
microencapsulated pomegranate	women with acute coronary syndrome	reverts high-density lipoprotein-induced endothelial dysfunction and ↓postprandial triglyceridemia	[96]

**Table 6:** Recent studies on the effect of the addition of pomegranate derivatives to dairy products.

Derivative	Product	Effect	References
juice	kefir-type	↑viscosity; ↑acidity	[97]
juice powder	yogurt	↑total phenolics; ↑antioxidant activity; ↑solid-like behavior	[98]
peel extract	cheese	↑lipid oxidative stability; ↑storage quality	[99]
peel extract powder	fermented milk	↑total phenolics; ↑antioxidant activity	[100]
peel extract powder	cheese	↑antioxidant activity; ↑shelf life	[101]
peel extract powder	freeze-dried yogurt	↑total phenolics; ↑antioxidant activity	[102]
seed powder	yogurt	↑antioxidant activity; fatty acid profile improvement	[103]

**Table 7:** Recent studies on the application of pomegranate derivatives to films and coatings.

Derivative	Product	Effect	References
peel extract	zein-based film (cheese)	↑tensile strength; ↑antioxidant and antimicrobial activity	[104]
peel extract	chitosan coating (rainbow trout, pacific white shrimp, strawberry)	improvement of functional characteristics of coatings; improvement of sensory characteristics and ↑shelf life of food	[105,106,107,108]
peel powder	starch-based films	better mechanical properties; antimicrobial activity	[109]
peel extract	chitosan/locust gum coating (oranges)	inhibition of *Penicillium digitatum*; ↑shelf-life of oranges	[110]
peel extract	surimi-based films	superior mechanical properties; improved thermal stability	[111]
juice	bitter vetch seed protein films	antioxidant activity; improvement of physicochemical properties	[112]

**Table 8:** Recent studies on the effect of the addition of pomegranate derivatives to meat and fish products.

Derivative	Product	Effect	References
peel powder	meatballs	↑antioxidant activity; ↓lipid and protein oxidation; ↑microbial quality	[113]
peel powder	beef sausage	quality criteria improvement; improvement of cooking characteristics	[114]
juice powder	raw ground chicken	↓heat resistance of *E. coli*	[115]
peel extract	pacific white shrimp	↓lipid oxidation; ↓melanosis; ↓microbial growth	[116]
extract	fish patties	↑shelf-life	[117]
extract	pork sausage	controlling microbial growth and oxidation; ↑shelf-life	[118]

**Table 9:** Recent studies on the effect of the addition of pomegranate derivatives to cereal and nut products.

Derivative	Product	Effect	References
peel extract	hazelnut paste	↑shelf life; delay oxidation	[119]
peel extract	cookies	↑shelf life; ↑antioxidant activity; ↑panelist acceptance (odor, color)	[120]
seed powder	gluten-free bread	↑specific volume and springiness; ↑antioxidant activity	[121]
seed powder	gluten-free cake	↑antioxidant activity, protein and fiber; ↓peroxide value	[122]
seed powder	gluten-free sheeted pasts	↑antioxidant activity; ↓cooking and textural parameters	[123]
